# Application of bioactive hydrogels combined with dental pulp stem cells for the repair of large gap peripheral nerve injuries

**DOI:** 10.1016/j.bioactmat.2020.08.028

**Published:** 2020-09-19

**Authors:** Lihua Luo, Yan He, Ling Jin, Yanni Zhang, Fernando P. Guastaldi, Abdullkhaleg A. Albashari, Fengting Hu, Xiaoyan Wang, Lei Wang, Jian Xiao, Lingli Li, Jianming Wang, Akon Higuchi, Qingsong Ye

**Affiliations:** a School and Hospital of Stomatology, Wenzhou Medical University, Wenzhou, Zhejiang, China; b Tianyou Hospital, Wuhan University of Science and Technology, Wuhan, 430064, China; c Skeletal Biology Research Center, Massachusetts General Hospital and Harvard School of Dental Medicine, Boston, MA, 02114, USA; d Wenzhou Institute of Biomaterials and Engineering, Wenzhou, Zhejiang Province, 325000, China; e Molecular Pharmacology Research Center, School of Pharmacy, Wenzhou Medical University, Wenzhou, 325035, China; f School of Ophthalmology and Optometry, Eye Hospital, Wenzhou Medical University, Wenzhou, Zhejiang, 325027, China; g Department of Biliary and Pancreatic Surgery/Cancer Research Center, Affiliated Tongji Hospital, Tongji Medical College, Huazhong University of Science and Technology, Wuhan, 430030, China; h Center of Regenerative Medicine, Renmin Hospital of Wuhan University, Wuhan, 430060, China

**Keywords:** Dental pulp stem cells, Human basic fibroblast growth factor, Gelatin methacrylate, Large gap, Peripheral nerve injuries, Nerve graft

## Abstract

Due to the limitations in autogenous nerve grafting or Schwann cell transplantation, large gap peripheral nerve injuries require a bridging strategy supported by nerve conduit. Cell based therapies provide a novel treatment for peripheral nerve injuries. In this study, we first experimented an optimal scaffold material synthesis protocol, from where we selected the 10% GFD formula (10% GelMA hydrogel, recombinant human basic fibroblast growth factor and dental pulp stem cells (DPSCs)) to fill a cellulose/soy protein isolate composite membrane (CSM) tube to construct a third generation of nerve regeneration conduit, CSM-GFD. Then this CSM-GFD conduit was applied to repair a 15-mm long defect of sciatic nerve in a rat model. After 12 week post implant surgery, at histologic level, we found CSM-GFD conduit could regenerate nerve tissue like neuron and Schwann like nerve cells and myelinated nerve fibers. At physical level, CSM-GFD achieved functional recovery assessed by a sciatic functional index study. In both levels, CSM-GFD performed like what gold standard, the nerve autograft, could do. Further, we unveiled that almost all newly formed nerve tissue at defect site was originated from the direct differentiation of exogeneous DPSCs in CSM-GFD. In conclusion, we claimed that this third-generation nerve regeneration conduit, CSM-GFD, could be a promising tissue engineering approach to replace the conventional nerve autograft to treat the large gap defect in peripheral nerve injuries.

## Highlights

• A novel 3^rd^ generation nerve conduit was successfully constructed and applied for repairing peripheral nerve injuries (PNI).
• Dental pulp stem cells (DPSCs) was optimized as an ideal seeding cells for nerve regeneration.
• A bioactive system combining GelMA with human bFGF and DPSCs could reconstruct the long gap PNI within 12 weeks in vivo.
• Our system could achieve the same outcome in nerve repair as that of autografting, a routine treatment for PNI.
• The proposed bioactive system may trigger an evolutional change into the current clinical practice in managing PNI.
• The majority of the regenerated nerve tissue was originated from the donor’s dental pulp stem cells.

## Graphical abstract

**Figure:**
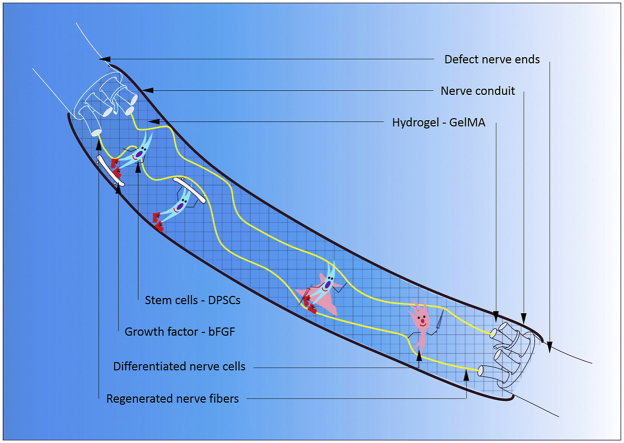

## 1 Introduction

Peripheral nerve injuries (PNIs) resulted from traffic accidents, natural disasters and military activity often lead to physical disability, chronic pain, loss of motor and sensory functions, and decreased life quality. Due to lack of necessary precursor cells and rapid apoptosis of neural cells, it is a challenge to repair damaged peripheral nerves [[1], [2], [3]]. Meanwhile, the length of nerve gap is a critical parameter affecting the success of peripheral nerve regeneration [4]. At transected site, if the gap is 0–1 cm, direct end to end neurorrhaphy is an effective method for the re-connection of injured nerve [5]. If the gap is larger than 1 cm, it requires nerve grafting or nerve conduit containing growth-stimulatory biomaterials. Autologous nerve grafting is regarded as the gold standard therapy for the long gap of peripheral nerve defects [6]. Yet its clinical application is restricted to the donor nerve availability, donor site morbidity, cost of secondary surgical procedure and morphometric matching [1,7]. In order to improve the success rate in repairing the large gap PNIs, alternative attempts are to engineer nerve conduit which composes of scaffold, growth factor, and stem cells [8,9].

Ideally an engineered nerve conduit should function as a bridge to concatenate proximal nerve defect to distal stump. Numerous synthetic and natural scaffolds, including poly-l-lactic acid [10], polylactic-co-glycolic acid copolymer [11], poly (l-lactide-co-6-caprolactone) [12], silk [13], collagen [14], fibronectin [15], and fibrin [14], have been explored as nerve conduits due to their excellent biodegradability, biocompatibility, and adjustable mechanical property [7,16]. As a type of scaffold with above properties, cellulose/soy protein isolate polymers have been extensively applied in peripheral nerve tissue engineering [5,7,17]. Our previous studies indicated that cellulose/soy protein isolate composite membrane (CSM) could be used as nerve conduits to bridge the stumps of injured nerve, as well as prohibit the invasion of surrounding connective tissue [7]. Due to insufficient seed cells and inappropriate extracellular matrix (ECM) in hollow nerve conduit, it is difficult achieve axonal regeneration to the same degree as autografting in large gap PNIs [18]. Synthetic ECM-like structure like hydrogel that possesses 3D porous structure and great cytocompatibility could to promote cellular adhesion, survival and proliferation [19]. Besides, hydrogel can be excellent vehicle for the delivery of biological factors (e.g. growth factors) which displayed a sustained-release profile [2]. Thus, novel hydrogels are necessary for nerve conduit in the repair of PNIs.

Nowadays, hydrogels derived from natural biomaterials, for example, the polysaccharide-based materials (such as cellulose, chitosan, etc) or the protein-based materials (such as collagen, gelatin, etc.), have been widely used in tissue engineering [20,21], because of their good biocompatibility and biodegradability, extensive availability and excellent biological properties [20]. Whereas, polysaccharide-based natural materials such as chitosan has poor solubility under physiological conditions, which is the key limiting factor for its application in tissue engineering [22,23]. Among protein-based materials, gelatin is a hydrolytic product of collagen but with lower immunogenicity. Gelatin has a good water-solubility and preserves abundant bioactive sequences such as the arginine-glycine-aspartic acid (RGD) which promotes the cell adhesion and growth as well as the expression of matrix metalloproteinases (MMPs). MMPs can enhance the cell remodeling and degradation [[24], [25], [26]]. Taken together, gelatin has been proven to be one of the ideal materials for extracellular matrix (ECM) mimic in tissue engineering and drug delivery system [27].

However, due to the poor thermostability and low melting point, gelatin is required for chemical modifications to overcome the shortcomings and avoid thermo reversibility [25,27]. When gelatin is chemically modified by methacryloyl (MA) then crosslinked by photoinitiator at a fixed wavelength of light, a gelatin methacryloyl (GelMA) hydrogel is synthesized with excellent thermostability [25,28,29]. Meanwhile, GelMA hydrogel keeps up the most of functional amino acid motifs (e.g. RGD and MMPs) because of its crosslinking is less than 5% [30]. GelMA hydrogel has been proven as an optimal biomaterial for tissue engineering and drug delivery due to its unique features such as polymerization under normal conditions, temporal and spatial control of reaction, tunable mechanical property and good biocompatibility [31,32]. Therefore, GelMA hydrogel is an ideal scaffold that can play dual roles in substituting ECM for cell attachment and controlling the release of growth factors for tissue engineering [25,27,33].

Schwann cells (SCs) are crucial components in endogenous repair of PNIs. SCs play an important role in reconstituting myelin and providing physical guidance for axonal regeneration [34,35]. However, the clinical application of autologous SCs is limited by lack of cell source and poor proliferation [36]. Therefore, a reliable source of SCs to ensure cell quantity and viability is essential for the peripheral nerve regeneration. Studies have confirmed that many mesenchymal stem cells (MSCs), e.g. bone marrow stromal cells and adipose-derived stem cells, can differentiate into Schwann-like cells to support the regeneration and re-myelination of injured axons in peripheral nerve repair [37,38]. The acquirement of these cells involves invasive surgical procedures like bone marrow aspiration and liposuction [34]. Dental pulp stem cells (DPSCs), as a kind of MSCs from dental pulp, can be obtained from impacted third molars and orthodontically extracted premolars, which does not cause extra harm or raise ethical concerns [2]. DPSCs, originating from the cranial neural crest, spontaneously express neural markers such as Nestin, β-tubulin III, and NeuN [39]. Studies have suggested that DPSCs can differentiate into Schwann-like cells to promote myelin reconstitution and axonal regeneration in PNIs [34,35]. Furthermore, DPSCs are reported to secrete several neurotrophic factors that provide neuroprotection and trophic support for axonal regeneration [2,40,41]. Therefore, DPSCs are a promising stem cell source for the repair of PNIs.

Fibroblast growth factor (FGF) has been verified to promote cell proliferation and survival in peripheral nerve tissue engineering [42]. Human basic fibroblast growth factor (bFGF), as a member of FGF family, is a critical neurotrophic factor that can promote cell proliferation, suppress apoptosis, and maintain stemness of MSCs in vitro [43]. Furthermore, bFGF has also been confirmed to enhance the proliferation and differentiation of endogenous neural progenitor cells [44]. Our previous studies have shown that bFGF accelerated the DPSCs proliferation and survival in vitro and combination of DPSCs with bFGF exhibited a much better neuronal regeneration than bFGF or DPSCs alone in repairing the central nerve injuries [45].

In this work, we fabricated the third-generation nerve conduit by filling CSM hollow nerve conduit with GelMA-bFGF hydrogels containing DPSCs, to bridge a 15-mm long gap of sciatic nerve defect in rat model. The aim of this study was to determine whether bFGF-loaded GelMA hydrogel could provide a continuously active microenvironment for DPSCs survival and proliferation and evaluate this novel third-generation of nerve conduits in the regeneration of large gap PNIs.

## 2 Materials and methods

### 2.1 Synthesis and analysis of gelatin methacrylate (GelMA)

GelMA was synthesized as previously described [46,47]. Briefly, 20 g of gelatin (type A, Sigma Aldrich, St. Louis, MO, USA) was dissolved in 200 mL Dulbecco's Phosphate Buffered Saline (DPBS, GIBCO, CA) at 50 °C under magnetic stirring condition. Then, 2 mL of methacrylic anhydride (MA, Sigma Aldrich, St. Louis, MO, USA) was added to the gelatin solution at a rate of 0.5 mL/min, allowing the reaction to proceed for 3 h. Finally, this GelMA mixture was dialyzed against deionized water using a 1 kDa dialysis tubing at 50 °C for five days with six water change every day. The final product was lyophilized by freeze dryer and stored at room temperature for further use.

The degree of methacrylation of GelMA was quantified by ^1^H-nuclear magnetic resonance (^1^H-NMR) spectrometer (Advanced III, 600 MHz, Bruker, Germany). Briefly, lyophilized GelMA foam and gelatin were dissolved in deuterium oxide (D_2_O) at 50 °C, respectively. Spectra were collected from the Advanced III ^1^H-NMR spectrometer and the peak area of lysine methylene protons was calculated in each spectrum. The degree of methacrylation was calculated as following equation [48]:

(1) $$The\;degree\;of\;methacrylation\;\left(\%\right)=\left(1-\frac{P_{GelMA}}{P_{gelatin}}\right)\;\times 100$$

where P_GelMA_ and P_gelatin_ were the peak area of lysine methylene protons in GelMA and gelatin at around 3.0 ppm, respectively.

### 2.2 Fabrication of GelMA-bFGF hydrogels

**Fig. 1:**
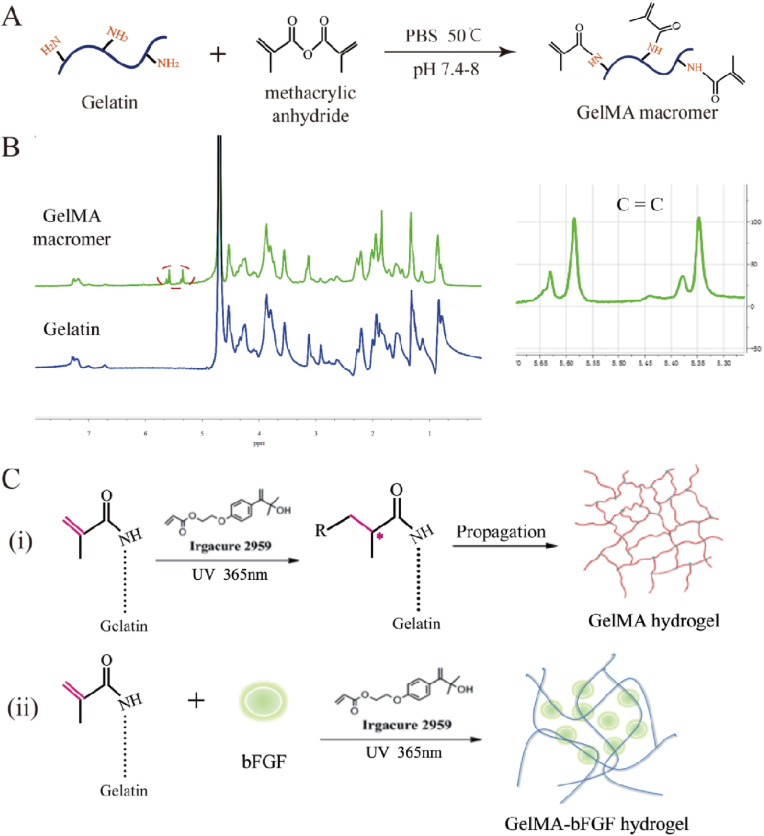
Synthesis and chemical compositions of GelMA macromer and GelMA/GelMA-bFGF hydrogels. (A) The preparation of GelMA macromer via the reaction of gelatin and methacrylic anhydride. (B) ^1^H NMR spectra of Gelatin and GelMA hydrogel. (C) GelMA and GelMA-bFGF hydrogels were fabricated by photopolymerization. (i) Free radicals originated from UV light-triggered initiators, which initiated GelMA hydrogel to form cross-linking networks; (ii) GelMA-bFGF hydrogel was prepared by mixing bFGF with GelMA macromer via photoploymerization.

Fig. 1C showed the synthesis of GelMA-bFGF hydrogels. Firstly, GelMA macromer was dissolved in PBS (pH = 7.0) at 50 °C to obtain GelMA solution at concentrations of 5, 10 and 15% (w/v). Secondly, 20 ng/mL of bFGF was added to these GelMA solutions with the modest stirring for 10 min at room temperature. Then, Irgacure 2959, a photo-initiator, was added to GelMA-bFGF solutions on 1:100 (v/v) ratio. The mixture was stirred for 5 min and aliquoted 100 μL per well in a 96-well plate. Finally, the plate was placed under UV light (365 nm, 10 mW/cm^2^) for 30 s to construct GelMA-bFGF hydrogels.

### 2.3 Fourier-transform infrared spectrometry (FT-IR) analysis

As for FT-IR, 5, 10 and 15% (w/v) GelMA/GelMA-bFGF hydrogels were freeze dried and grounded into fine powder and then pelletized with KBr to form a transparent sheet. The spectra were recorded on the FT-IR spectrometer (1600, PerkinElmer Co., Boston, MA, USA) in the wavenumber ranged from 4000 to 400 cm^−1^.

### 2.4 Scanning electron microscopy (SEM)

The morphology and pore structure of GelMA and GelMA-bFGF hydrogels were observed by scanning electron microscopy (SEM, H-7500, Hitachi, Japan). GelMA and GelMA-bFGF hydrogels were placed on copper meshes, flash frozen in liquid nitrogen and lyophilized by vacuum freeze dryer overnight. The specimen surface was then coated with gold/palladium (Au/Pd) for SEM observation. The average pore size was calculated from SEM images through the high-resolution imaging treatment system (HLPAS-1000, Wenzhou Medical University, Wenzhou, China).

### 2.5 Swelling ability

The swelling property of GelMA-bFGF hydrogels was investigated by conventional gravimetric method. Briefly, lyophilized GelMA-bFGF hydrogels (5, 10 and 15% (w/v)) were immersed in PBS at 37 °C for pre-determined time intervals. The initial weight of dry GelMA-bFGF hydrogels before incubation, recorded as W_0_. At designated time point, GelMA-bFGF hydrogels were taken out of PBS to measure the weight, recorded as W_1_. Prior to weighing, hydrogel was placed between two filter paper to gently removed excess liquid. Then, hydrogel was placed back in PBS. The swelling rate was calculated as following equation [46]:

(2) $$The\;swelling\;rate\;\left(\%\right)=\frac{W_{1}-W_{0}}{W_{0}}\times 100$$

### 2.6 Mechanical characterization

The mechanical property of GelMA-bFGF hydrogels (5, 10 and 15% (w/v)) was evaluated by a DHR-1 rheometer (TA Instruments, USA). The storage modulus (G′) of GelMA-bFGF hydrogels were tested in the oscillatory mode using a parallel configuration (8 mm in diameter) at 37 °C. The gap between two plates was set at 1.0 mm. Subject to a fixed strain of 0.1%, the storage modulus (G’) and the loss modulus (G”) of GelMA-bFGF hydrogels were measured by dynamic frequency sweep test at a frequency range of 0.01–10 Hz. All tests were performed in triplicate.

### 2.7 bFGF release profile

In vitro release profile of bFGF from GelMA-bFGF hydrogels (5%, 10% and 15% (w/v)) was evaluated by an enzyme-linked immunosorbent assay (ELISA) method. Briefly, 100 μL of GelMA-bFGF hydrogels containing 100 ng of bFGF was immersed in 500 μL of PBS and placed on a rotary shaker (50 rpm) at 37 °C. At established time points (1, 5, 7, 12 h and 3, 5, 7, 14, 21, 28 days), the solutions were centrifuged (1500 g), 37 °C, 5 min. Supernatants were collected for ELISA and 500 μL fresh PBS was added. bFGF concentrations of the above supernatants were quantified by bFGF ELISA kits (Westang System, Shanghai, China) according to manufacturer's instructions.

### 2.8 Degradation analysis

The in vitro degradation rate of GelMA-bFGF hydrogels was determined as previously described [46]. Briefly, lyophilized GelMA-bFGF hydrogels (5, 10 and 15% (w/v)) were weighed (W_0_) and incubated in PBS supplemented with 1 ng/mL of collagenase type I (Gibco, USA). All samples were kept in the shaker incubator (150 rpm) for 1, 3, 5, 7, 14, and 21 days at 37 °C. The liquid was replaced every day. At determined time points, hydrogels were picked out for lyophilization and weighed (W_1_). The degradation rate was calculated using equation [46]:

(3) $$The\;degradation\;rate\;\left(\%\right)=\;\frac{W_{1}-\;W_{0}}{W_{0}}\;\times 100$$

The in vivo degradation of GelMA-bFGF hydrogels (5, 10 and 15% (w/v)) was analyzed by subcutaneous and muscle implantation. 24 Sprague-Dawley (SD) male rats, weighing 200–250 g, were obtained from the Animal Center of Chinese Academy of Science (Shanghai, China) and acclimatized in the animal care facility for two weeks prior to surgery. The animals were randomly divided into three groups (8 rats per group), including 5%, 10% and 15% GelMA-bFGF groups. In each group, subcutaneous and muscle implantations were randomly performed with four animals per implantation. After anesthesia, the implantation area was shaved, marked and sterilized prior to surgery. For subcutaneous implantation, two small bilateral subcutaneous pockets (1 mm × 1 mm right angle incision) were prepared along the backbone. Then one GelMA-bFGF hydrogel block per pocket was implanted into subcutaneous pockets. For muscle implantation, about 1 mm skin incision was made at the lateral calf, one GelMA-bFGF hydrogel block per leg was implanted into the middle of ischial gastrocnemius. Then, the skin and muscle of all incisions were closed with 5-0 interrupted absorbable sutures. 30 days post implantation, the animals were sacrificed, and the specimens were harvested for hematoxylin-eosin (HE) staining. The use of rats and protocols in this study were independently reviewed and approved by the Ethics Committee of the School and Hospital of Stomatology, Wenzhou Medical University (No. WYKQ2018008AE). All experiments were performed according to the Guide of Chinese National Institutes of Health and the Animal Care and Use Committee of Wenzhou Medical University.

### 2.9 Isolation, culture and identification of DPSCs

The isolation, culture and identification of DPSCs were described in our previous work [45]. Briefly, dental pulp tissue was removed from impacted third molars, which were collected from the Stomatology Hospital of Wenzhou Medical University, Wenzhou, China. Then, pulp tissue was cut into small pieces and digested with dispase (Sigma, Germany) and collagenase type I for 30 min at 37 °C. The cellular suspension was placed in T-25 culture flask and incubated with α-modified Eagle's medium (α-MEM, Gibco, USA) containing 20% fetal bovine serum (FBS, Gibco, USA), 100 μg/mL streptomycin, and 100 U/mL penicillin (Gibco, USA) in 5% CO_2_ at 37 °C. The culture medium was changed every 3 days. The stemness of DPSCs was identified by flow cytometry using the primary antibodies of human CD73 (BD Pharmingen™, USA), CD90 (BD Pharmingen™, USA), CD14 (BioLegend, USA), and HLA-DR (BioLegend, USA). The data were analyzed by CytoFLEX flow cytometers (Beckman Coulter, California, USA). The multipotent characteristic of DPSCs was analyzed by osteogenic, adipogenic, and chondrogenic differentiation according to the methods described in our previous study [45]. OriCellTM MSC Differentiation Medium (Cyagen, Santa Clara, CA) was used for the differentiation studies. And the procedure of differentiation was performed according to the manufacturer's instructions. At the end of differentiation, the ability of osteogenic, adipogenic, and chondrogenic differentiation was evaluated by Alizarin Red S staining, Oil Red O staining, and Alcian Blue staining, respectively. Images were taken and analyzed using light microscopy (TS100, Nikon). The use of DPSCs and protocols described in this study were independently reviewed and approved by the Ethics Committee of the School and Hospital of Stomatology, Wenzhou Medical University (No. WYKQ2018008SC).

### 2.10 DPSCs encapsulation, viability, proliferation, and immunofluorescence analyses

For the construction of GelMA-bFGF hydrogels loaded with DPSCs, DPSCs (passage 3) were collected and re-suspended in GelMA-bFGF solutions (5, 10 and 15% (w/v)) to yield a final density of 1 × 10^6^ cells/mL. Then the mixture was added to a 96-well plate and photo-cross-linked to obtain GelMA-bFGF-DPSCs (GFD) hydrogels. The viability of DPSCs in GelMA-bFGF hydrogels was analyzed by the Live/Dead Viability/Cytotoxicity Kit (Invitrogen, CA, USA). At each pre-determined time points, randomly selected areas of each sample were observed and photographed by fluorescence microscope (Axiovert A1, Carl Zeiss, Germany) and the number of live and dead cells were calculated using Image J.

As for the proliferation analysis, a CCK-8 assay (Dojindo, Kumamoto, Japan) was performed to evaluate the survival of DPSCs in GelMA-bFGF hydrogels. The GFD samples were added to 96-well plate and cultured in 5% CO_2_ at 37 °C_._ After 1, 3, 5, 7, and 9 days, CCK-8 solution was added and incubated for 1 h. OD values of CCK-8 solution were measured at 450 nm with an absorbance microplate reader (Varioskan LUX, Thermo Fisher, USA). Meanwhile, on day 5, the cells were stained by immunofluorescence labels, glial fibrillary acidic protein (GFAP) and β-tubulin III (Sigma-Aldrich, USA), and observed by fluorescence microscope.

### 2.11 Fabrication of CSM nerve conduit combined with GFD hydrogels

Cellulose/soy protein isolate composite membrane (CSM) was fabricated as described in our previous work [7]. Briefly, NaOH/urea aqueous solution was used to dissolve cellulose at −12 °C and soy protein isolate at room temperature to obtain cellulose solution and soy protein isolate solution, respectively. Then the soy protein isolate solution was added dropwise into cellulose solution at 4 °C and stirred for 30 min to get the cellulose/soy protein isolate mixture, which contained 30 wt% of soy protein isolate. After centrifugation, the mixture solution was cast on a glass plate and coagulated in 5 wt% acetic acid aqueous solution to obtain a transparent membrane. Air-dried CSM was used in this study.

For the preparation of nerve conduit, CSM was cut into appropriate size, rolled and stitched with a 8-0 absorbable thread to obtain a hollow tube to bridge the nerve gap and to guide the axonal regeneration (Fig. A2A). The GFD solution that contained Irgacure 2959 were added to fill in the CSM tube. Then photo-cross-linking was performed by UV light (365 nm, 10 mW/cm^2^) for 30 s to construct the CSM-GFD nerve conduit (Fig. A2B), which was incubated at 37 °C for 12 h before use. All procedures were performed aseptically.

### 2.12 Surgical procedure

36 Sprague-Dawley male rats, weighing 200–250 g, were purchased from the Animal Center of Chinese Academy of Science (Shanghai, China) and acclimatized in the animal care facility of Wenzhou Medical University for two weeks before surgery. The animals were randomly divided into sham control group (n = 6) and three experimental groups including nerve autograft (NA) group (n = 10), CSM nerve conduit filled with GelMA hydrogel (CSM-G) group (n = 10), and CSM nerve conduit filled with GFD hydrogel (CSM-GFD) group (n = 10). Animals were anesthetized by an intraperitoneal injection of 10% chloralic hydras at a dose of 3.5 mL per kg body weight. After anesthesia, the hair of lateral thigh was shaved and the skin was sanitized with 70% alcohol. Skin incision extended from the lateral femoral oblique and muscles were split to expose the sciatic nerve. In NA group, 13 mm nerve fiber was resected, flipped 180° longitudinally, and sutured back to bridge the nerve gap. In CSM-G and CSM-GFD groups, 13 mm nerve fiber was removed to make a 15 mm nerve gap. Then, CSM nerve conduit filled with GelMA hydrogel and CSM nerve conduit filled with GFD hydrogel were used to bridge this 15 mm nerve gap by 8-0 nylon sutures (PGA, Jinhuan, Shanghai), respectively. The sham control group received the same surgical procedures to expose the nerve, but the sciatic nerve was not damaged. The muscle and skin of all animals were closed with 5-0 interrupted absorbable sutures (PGA, Jinhuan, Shanghai). Postoperative animals were conventionally housed. The use of rats and protocols in this study were independently reviewed and approved by the Ethics Committee of the School and Hospital of Stomatology, Wenzhou Medical University (No. WYKQ2018008). All experimental procedures were performed according to the Guide of Chinese National Institutes of Health and the Animal Care and Use Committee of Wenzhou Medical University.

### 2.13 Walking track analysis

12 weeks after surgery, walking track analysis was performed to evaluate the motor function of animals. Hind claws of the animal were dipped in red ink and the animal was released to walk through a flat track lined with paper to record the hind footprints. From the footprint pattern, sciatic functional index (SFI) was calculated as following equation [7]:

(4) $$\text{SFI}=\left(-38.3\times \frac{EPL-NPL}{NPL}\right)+\left(109.5\times \frac{ETS-NTS}{NTS}\right)+\left(13.3\times \frac{EIT-NIT}{NIT}\right)-8.8$$

where print length (PL) measured the distance from the heel to the third toe, toe spread (TS) measured the distance from the first to the fifth toe, and intermediary toe spread (IT) measured the distance from the second to the fourth toe were measured. Prefix E and N represented the defected paw and normal paw, respectively.

### 2.14 Histological analysis

As for histology evaluation, animals were euthanized and sciatic nerve was dissected after 12 weeks post implantation. Nerve specimens were fixed with 4% paraformaldehyde for 6 h, dehydrated with ethanol and embedded in paraffin. Then, the samples were cut into 5 μm sections, stained with hematoxylin-eosin (HE), and observed by light microscope (TS100, Nikon, Japan). The regeneration of axon and myelin sheath in sciatic nerve were evaluated by toluidine blue staining. After euthanization, the nerve segments were picked out and fixed with 2.5% glutaraldehyde 2 h, then fixed with 1% osmium tetroxide for 2 h, dehydrated by ethanol and embedded in Epon812. Semi-thin sections were obtained using a glass knife, stained with toluidine blue and observed by light microscope (TS100, Nikon, Japan).

### 2.15 Immunohistochemical analysis

For the immunohistochemical analysis, paraffin-embedded sections were dewaxed, hydrated, and blocked with 5% bovine serum albumin (SW3015, Solarbio) for 30 min. Then, the sections were incubated with the following primary antibodies at 4 °C overnight: rabbit polyclonal anti-rat/human myelin basic protein (MBP) (1:200, 78896; Cell Signaling Technology, Danvers, MA, USA), rabbit polyclonal anti-rat/human S100 (1:200, ab868; Abcam, Cambridge, UK), mouse monoclonal anti-rat/human GFAP (1:400, G6171; Sigma-Aldrich, St. Louis, MO, USA), rabbit monoclonal anti-human MBP (1:400, ab133620; Abcam, Cambridge, UK), rabbit polyclonal anti-human S100 (1:300, ab15520; Abcam, Cambridge, UK), and mouse monoclonal anti-human GFAP (1:400, ab8975; Abcam, Cambridge, UK). Subsequently, the sections were rinsed with PBS and incubated with horseradish peroxidase conjugated goat anti-mouse or anti-rabbit secondary antibodies for another 2 h at 37 °C. Finally, the sections were stained with a DAB (3,3′-diaminoben zidine) Kit (Zsbio, Beijing, China) and observed by fluorescence microscope (Axiovert A1, Carl Zeiss, Germany). Areas of positive staining were determined using the Image J software.

### 2.16 Western blot analysis

At 12 weeks after implantation, the nerve segments with conduits plus a ~15 mm portion from the proximal and distal stumps were harvested for Western blot analysis. Animals were euthanized, and nerve specimens were collected and stored at −80 °C as soon as possible. Then the nerve samples were homogenized in modified RIPA buffer with protease inhibitor cocktail (GE Healthcare Biosciences, PA, USA) and centrifuged at 12,000 rpm to obtain the supernatants for protein analysis. The extracts were quantified using a BCA Assay Kit (Beyotime, Shanghai, China). Briefly, proteins (30 μg) were added to electrophoresis in SDS-PAGE gels, and then transferred onto the PVDF membranes (Millipore, Germany). After blocking nonspecific binding sites, the membranes were incubated with the primary antibodies of rabbit polyclonal anti-rat/human MBP (1:1000, 78896; Cell Signaling Technology, Danvers, MA, USA), rabbit polyclonal anti-rat/human S100 (1:1000, ab868; Abcam, Cambridge, UK), mouse monoclonal anti-rat/human GFAP (1:5000, G6171; Sigma-Aldrich, St. Louis, MO, USA), rabbit monoclonal anti-human MBP (1:10000, ab133620; Abcam, Cambridge, UK), mouse monoclonal anti-human S100 (1:1000, sc-52206; Santa Cruz Biotechnology, Santa Cruz, CA, USA), and mouse monoclonal anti-human GFAP (1:500, ab8975; Abcam, Cambridge, UK) at 4 °C for 16 h. Subsequently, the membranes were incubated with the horseradish peroxidase-conjugated secondary antibodies for another 1 h at room temperature. Target proteins were analyzed by ChemiDoc XRS chemiluminescence imaging system (Bio-Rad, Hercules, CA, USA).

### 2.17 Statistical analysis

All quantitative data were presented as mean ± standard deviation (SD). Statistical differences were performed by Student's *t* test and one-way analysis of variance (ANOVA), followed by Tukey's test or Dunnett *post hoc* test. *P* < 0.05 was considered as the differences being statistically significant. Statistical analysis was performed by SPSS 19.0 (SPSS, Chicago, IL).

## 3 Results

### 3.1 Synthesis and characterization of GelMA macromer

In this study, gelatin was methacrylated through the reaction with methacrylate anhydride (MA) and supplemented with Irgacure 2959, a photoinitiator, to be photo curable at certain wavelength (Fig. 1A). The chemical composition of methacrylated gelatin (GelMA) was analyzed by ^1^H-NMR. As shown in ^1^H-NMR spectrum (Fig. 1B), new resonance peaks at 5.34 and 5.60 ppm were attributed to the presence of protons in H_2_C=C(CH_3_)- of GelMA, in contrast to no chemical signals appearing at 5.2–6.0 ppm in gelatin, which was consistent with previously study [49]. ^1^H-NMR results confirmed the successful methacrylation of gelatin. Also, according to the Habeeb method [48], the degree of substitution in GelMA calculated from ^1^H-NMR was 51.0%.

**Fig. 2:**
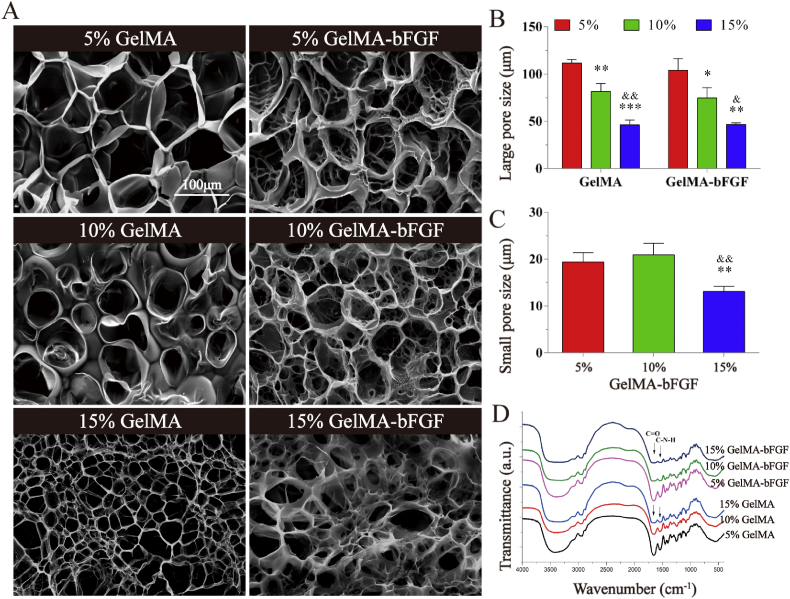
SEM images and FT-IR spectra of GelMA and GelMA-bFGF hydrogels. (A) The morphology of GelMA and GelMA-bFGF hydrogels. (B) The large pore size of GelMA and GelMA-bFGF hydrogels. (C) The small pore size of GelMA-bFGF hydrogels. (D) The FT-IR spectra of GelMA and GelMA-bFGF hydrogels. All data were represented as mean ± SD, ^∗^*P* < 0.05, ^∗∗^*P* < 0.01, ^∗∗∗^*P* < 0.001 versus 5% GelMA in the hydrogel groups, ^&^*P* < 0.05, ^&&^*P* < 0.01 versus 10% GelMA in the hydrogel groups.

### 3.2 Morphology and FT-IR of GelMA and GelMA-bFGF hydrogels

In this study, the microstructure of GelMA and GelMA-bFGF hydrogels was observed and compared by SEM. As shown in Fig. 2A, the results revealed that GelMA and GelMA-bFGF hydrogels both possessed a large pore surface morphology. And the size of large pore in both hydrogels noticeably decreased with an increase of GelMA content in the hydrogels (P < 0.05) (Fig. 2B). Additionally, compared to GelMA hydrogels, GelMA-bFGF hydrogels also contained lots of internally connected small pores, which had sizes ranged from 13.10 ± 1.09 μm to 20.89 ± 2.50 μm. The size of small pore in 15% GelMA-bFGF hydrogel was significantly smaller than that in 5% and 15% GelMA-bFGF hydrogels (Fig. 2C). In FT-IR spectra of GelMA and GelMA-bFGF as shown in Fig. 2D, the characteristic peaks such as C=O and C-N-H bands had no difference between GelMA and GelMA-bFGF hydrogels.

### 3.3 Swelling ratios, mechanical characteristics, and bFGF release profile of GelMA-bFGF hydrogels

**Fig. 3:**
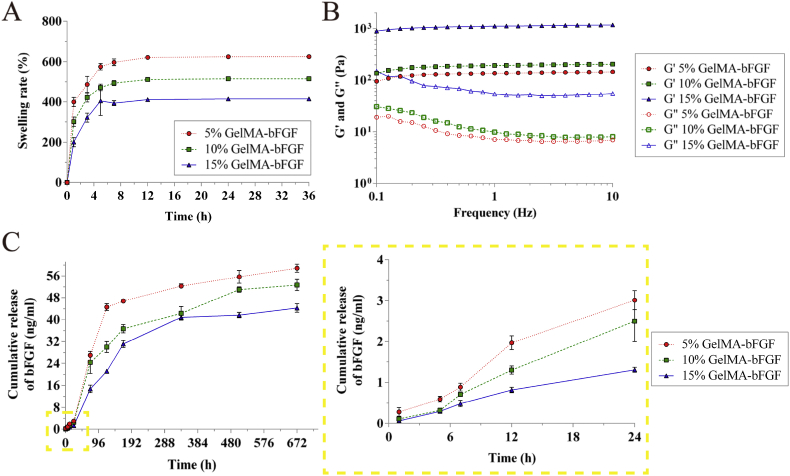
Swelling ratio, mechanical property and bFGF release profile of GelMA-bFGF hydrogels. (A) The degree of swelling of GelMA-bFGF hydrogels. (B) Frequency sweep test of GelMA-bFGF hydrogels, elastic (storage) modulus G′ and the viscous (loss) modulus G″ at the strain rate of 0.1%. (C) The bFGF release profile of GelMA-bFGF hydrogels in 28 days and a zoom in graph of release in the first 24 h.

As demonstrated in Fig. 3A, the swelling ratio of hydrogels had a dramatic increase in the first 5 h, and then gradually approached a plateau. The degree of swelling decreased with the increase of the proportion of GelMA macromer in hydrogels, which reached a balanced point after 12 h at 621.30 ± 6.12%, 511.18 ± 6.44% and 411.09 ± 6.34% in 5%, 10% and 15% GelMA-bFGF hydrogels, respectively.

As for the mechanical characteristics of GelMA-bFGF hydrogels, the elastic (storage) modulus G′ and the viscous (loss) modulus G″ were investigated by dynamic mechanical analysis, which provided quantitative information by measuring the mechanical response of the hydrogels under a periodic strain challenge. In Fig. 3B, the results indicated that the elastic modulus (G′) was superior to the viscous modulus (G″) in all concentration groups of GelMA-bFGF hydrogels. This suggested that GelMA-bFGF hydrogels had a predominantly elastic rather than viscous character. Moreover, both G′ and G″ apparently increased with the increase of GelMA macromer, where 15% GelMA-bFGF hydrogel showed the highest G′ and G′′.

GelMA hydrogels had the ability to form polyionic complex with growth factors, which resulted in a sustained release of growth factors. The release profile of bFGF in GelMA-bFGF hydrogels suggested that a similar cumulative release profile was found in GelMA-bFGF hydrogels of all concentration groups (Fig. 3C). A burst release of bFGF was observed within the first 5 days: 44.6 ± 1.25% in 5% GelMA hydrogel, 30.0 ± 2.00% in 10% GelMA hydrogel and 21.1 ± 0.46% in 15% GelMA hydrogel. After 28 days, the release of bFGF was 58.83 ± 1.46%, 52.67 ± 2.08% and 44.27 ± 1.54% in 5%, 10% and 15% GelMA-bFGF hydrogels, respectively. The release rate of bFGF decreased as GelMA macromer concentration increased in the hydrogels.

### 3.4 In vitro and in vivo degradability of GelMA-bFGF hydrogels

**Fig. 4:**
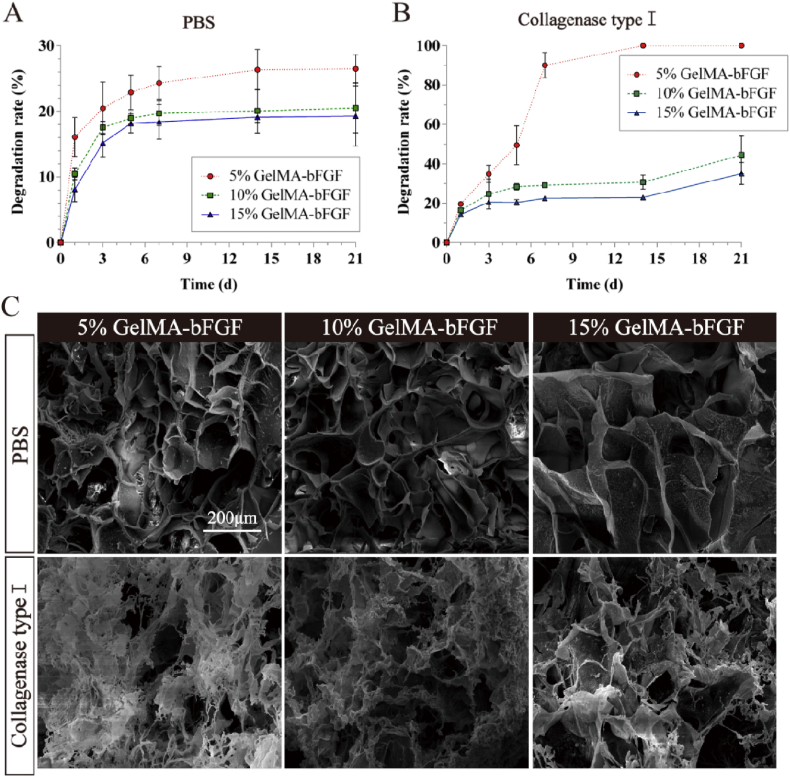
The in vitro degradability of GelMA-bFGF hydrogels. (A&B) Degradation profile of GelMA-bFGF hydrogels incubated in pure PBS and collagenase type I at 37 °C shaker (150 rpm) for 21 days. (C) SEM image of GelMA-bFGF hydrogels after 14 day degradation in PBS and collagenase type I.

The in vitro degradation profile of GelMA-bFGF hydrogels in PBS with and without collagenase was demonstrated in Fig. 4. The degradation rate of GelMA-bFGF hydrogels increased as the decrease of GelMA macromer in the hydrogels. On day 21, less than 30% of the hydrogel of all three types of hydrogels has degraded in PBS (Fig. 4A), much less than that of in collagenase.

In collagenase supplemented PBS, 5% GelMA-bFGF started with a fast degradation and half of the hydrogel had degraded on day 5. Degradation plateau was reached on day 7, by when approximately 90% of the hydrogel had degraded. On day 14, the 5% hydrogel was completely degraded (Fig. 4B). In contrast to no complete degradation of hydrogel in PBS, 10% and 15% GelMA hydrogels had reached the degradation plateau on day 5 and demonstrated a moderate degradation profile in collagenase (Fig. 4A and B). Compared with 5% GelMA-bFGF hydrogel, 10% and 15% GelMA-bFGF hydrogels were more resistant to enzymatic degradation, and the weight loss were 44.54 ± 9.53% and 35.13 ± 5.54% after 21 days (Fig. 4B).

The morphology of GelMA-bFGF hydrogels in degradation on day 14 were observed via SEM, shown in Fig. 4C. GelMA-bFGF hydrogels incubating in PBS showed no significant degradation and maintained its original pore structures, whereas the hydrogels obviously degraded in collagenase environment. Obviously, in 5% and 10% GelMA-bFGF hydrogels, substantial part of the hydrogel became fine and delicate; 3D porous construct was lost as the structure collapsed. The results were consistent with the degradation profiles quantified in Fig. 4A and B.

**Fig. 5:**
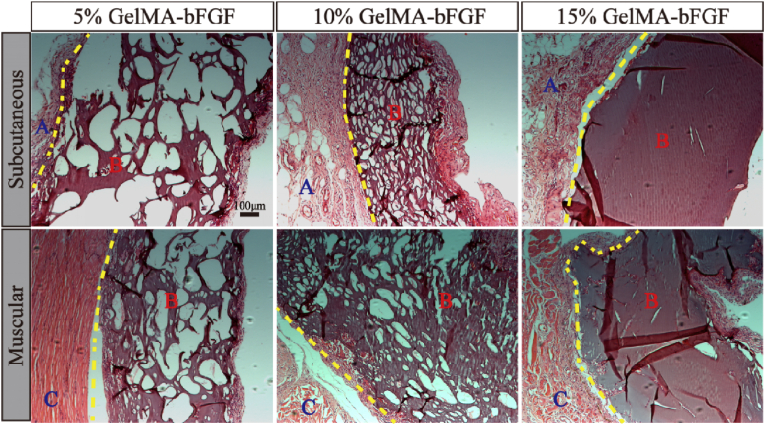
The HE images of GelMA-bFGF hydrogels implanted in subcutaneous and ischial gastrocnemius for 30 days. Yellow line: the boundary line of tissue and hydrogel; A: fibrous tissue; B: hydrogel; C: muscle. (For interpretation of the references to colour in this figure legend, the reader is referred to the Web version of this article.)

Histological evaluation of the in vivo degradation of GelMA-bFGF hydrogels was done on day 30 post implantation (Fig. 5). Both subcutaneous and muscular implantations of GelMA-bFGF hydrogels shown various levels of degradation and surrounding tissue response. HE photographs indicated that all hydrogel implants were encapsulated by fibrous tissue, which contained inflammatory cells and neovascular elements. At both locations, the hydrogels demonstrated a decreasing degradation in the order of 5%, 10% and 15% GelMA-bFGF. 5% GelMA-bFGF hydrogel at both sites displayed large voids and highly porous structure with the subcutaneous site being more obvious. 10% GelMA-bFGF hydrogel of both sites overlaid with small to medium voids and scattered a few large ones. There weres a few voids on the dense hydrogel background observed in 15% GelMA-bFGF hydrogel of both sites. The results indicated that the in vivo degradation rate of the hydrogels increased with the decreasing GelMA content in the hydrogels, consistent with the in vitro degradation data (Fig. 4).

### 3.5 Biocompatibility of GelMA-bFGF hydrogels with DPSCs

**Fig. 6:**
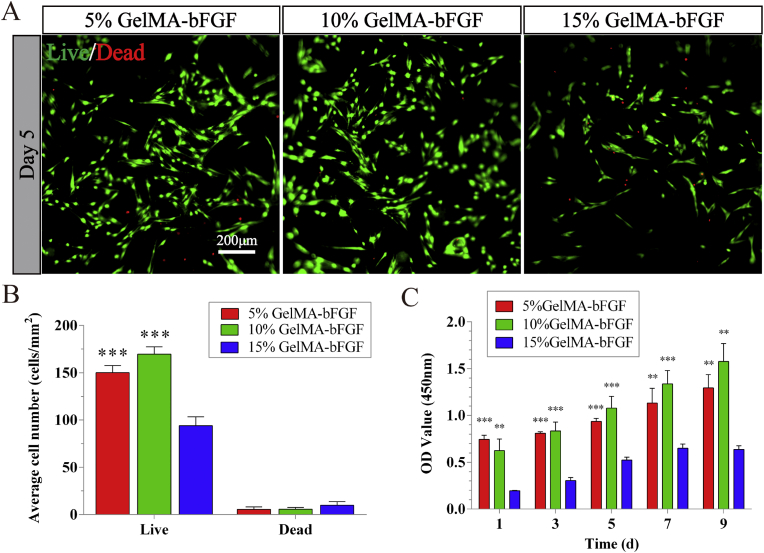
The viability and proliferation of DPSCs co-cultured with GelMA-bFGF hydrogels. (A) Live/Dead staining of DPSCs in GelMA-bFGF hydrogels at 5 days. (B) Quantification of the cells in GelMA-bFGF hydrogels by Image J. (C) The cell proliferation of DPSCs in GelMA-bFGF hydrogels at day 1, 3, 5, 7, and 9. Data were displayed in mean ± standard error from 3 replicates in each group. ^∗∗^*P* < 0.01, ^∗∗∗^*P* < 0.001 versus the 15% GelMA-bFGF group.

DPSCs, being one kind of MSCs, had great proliferation (Fig. A1A-C) and positively expressed MSCs-like markers such as CD73 and CD90 (Fig. A1D). Meanwhile, DPSCs had great multilineage differentiation potential, which had capacity to be induced to osteogenic, adipogenic, and chondrogenic differentiations (Fig. A1E-G). The viability of encapsulated DPSCs in GelMA-bFGF hydrogels was analyzed by Live/Dead assay. As shown in Fig. 6A, Live/Dead assay results indicated that 5% and 10% GelMA-bFGF hydrogels had good cytocompatibility with DPSCs. Lots of DPSCs were alive, stained in green. In addition, the number of viable DPSCs in 5% and 10% GelMA-bFGF hydrogels was higher than that of in 15% GelMA-bFGF hydrogels (P < 0.05). The number of dead cells, stained in red, had no obvious difference among three hydrogels (Fig. 6B).

The proliferation of encapsulated DPSCs in GelMA-bFGF hydrogels was evaluated for 9 days by CCK-8 assay (Fig. 6C). The results showed that DPSCs continued proliferating in three concentrations of GelMA-bFGF hydrogels. The cellular proliferation in 5% and 10% GelMA-bFGF hydrogels was much higher than that of in 15% GelMA-bFGF hydrogel (P < 0.05). In addition, from day 5 to day 9, the cellular proliferation rate of 10% GelMA-bFGF hydrogels reached the highest level compared to the rest hydrogels.

**Fig. 7:**
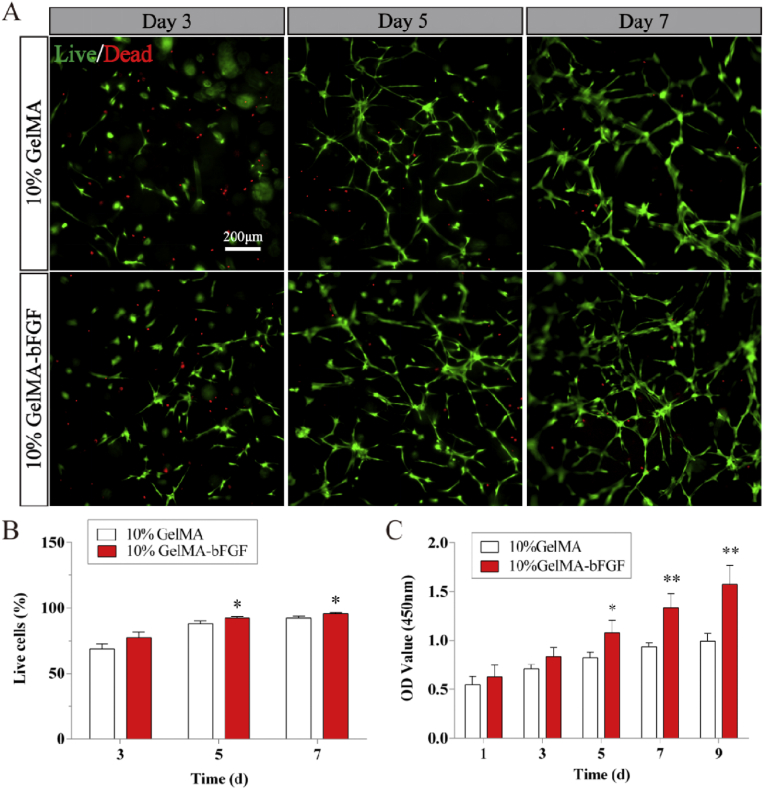
The viability and proliferation of DPSCs cultured with pure GelMA and 10% GelMA-bFGF hydrogels. (A) Live/Dead staining of DPSCs in pure GelMA and 10% GelMA-bFGF hydrogels at day 3, 5, and 7. (B) Quantification of live cells in pure GelMA and 10% GelMA-bFGF hydrogels by Image J. (C) The cell proliferation of DPSCs in pure GelMA and 10% GelMA-bFGF hydrogels at day 1, 3, 5, 7, and 9. Data were displayed in mean ± standard error from 3 replicates in each group. ^∗^*P* < 0.05, ^∗∗^*P* < 0.01 versus the 10% GelMA group.

As for the applications in nerve tissue engineering, the biomaterial should have the suitable pore size, mechanical property, and good biocompatibility and biodegradability. According to the above results, 10% GelMA-bFGF hydrogel had shown appropriate physical characteristics and biological properties. In the following experiment, we chose this hydrogel as the scaffold in the assessment of nerve regeneration. Compared to 10% pure GelMA hydrogel, 10% GelMA-bFGF hydrogel provided a suitable microenvironment for DPSCs survival in 3D culture (Fig. 7). The morphology of DPSCs gradually elongated from day 3 to day 7 (Fig. 7A). In addition, the number of viable DPSCs had obvious difference between 10% pure GelMA and 10% GelMA-bFGF hydrogels at day 5 and day 7 (P < 0.05) (Fig. 7B). CCK-8 assay suggested that being encapsulated in the hydrogels, DPSCs had good proliferation and the number of DPSCs gradually increased from day 1 to day 9. The proliferation rate of DPSCs in 10% GelMA-bFGF hydrogel was higher than that of in 10% pure GelMA hydrogel with a statistic significance since day 5 (P < 0.05) (Fig. 7C).

**Fig. 8:**
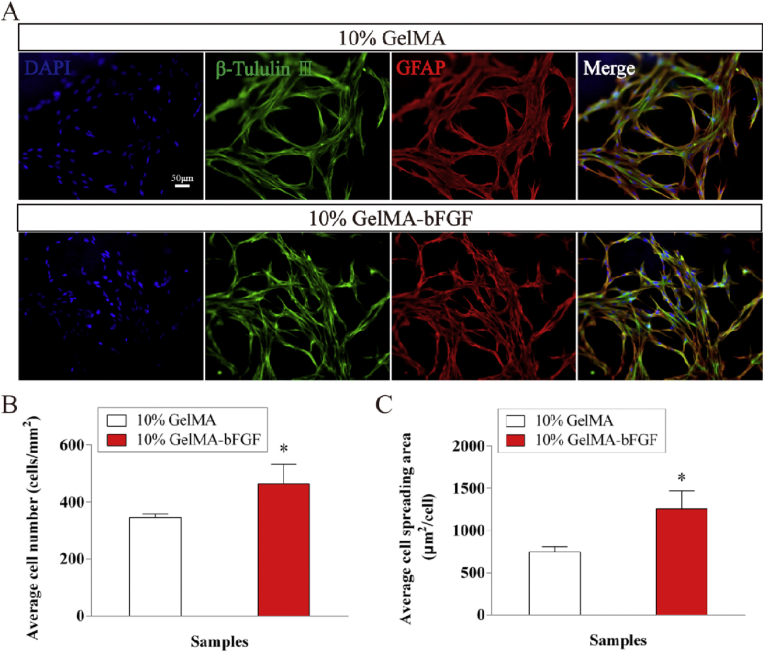
The immunofluorescent microscopy images of DPSCs cultured with pure GelMA and 10% GelMA-bFGF hydrogels. (A) The expression of GFAP and β-tubulin III after DPSCs co-cultured with the pure GelMA and 10% GelMA-bFGF hydrogels at 5 days. (B) Quantification of the GFAP and β-tubulin III positive expression of DPSCs in pure GelMA and 10% GelMA-bFGF hydrogels. (C) The spreading area of DPSCs in pure GelMA and 10% GelMA-bFGF hydrogels. Data were displayed in mean ± standard error from 3 replicates in each group. ^∗^*P* < 0.05 versus the 10% GelMA group.

Immunofluorescence staining results indicated that when co-cultured with both hydrogels, DPSCs positively expressed the neural surface markers such as GFAP and β-tubulin III (Fig. 8A). DPSCs evenly spread through the hydrogel outlining the porous structure of hydrogels. The number of DPSCs in 10% GelMA-bFGF hydrogel was significantly higher than that of in 10% pure GelMA hydrogel (Fig. 8B). Moreover, the spreading area of DPSCs in 10% GelMA-bFGF hydrogel was obviously bigger than that of in 10% pure GelMA hydrogel (Fig. 8C). Therefore, 10% GelMA-bFGF hydrogel could become a promising scaffold biomaterial for the repair and regeneration of peripheral nerve injuries.

### 3.6 Motor function evaluation

The recovery of motor function of the experimental animals was analyzed by SFI study and the results were shown in Fig. 11C. The results indicated that the control group showed the highest SFI value (P < 0.05), and the SFI score decreased in following order: control group (−8.043 ± 2.222), NA group (−28.591 ± 3.620), CSM-GFD group (−36.902 ± 5.214), and CSM-G group (−60.877 ± 4.317). Comparing with the gold standard, NA group, CSM-GFD group demonstrated a comparable nerve regeneration scored by SFI (P > 0.05).

### 3.7 HE staining analysis

**Fig. 9:**
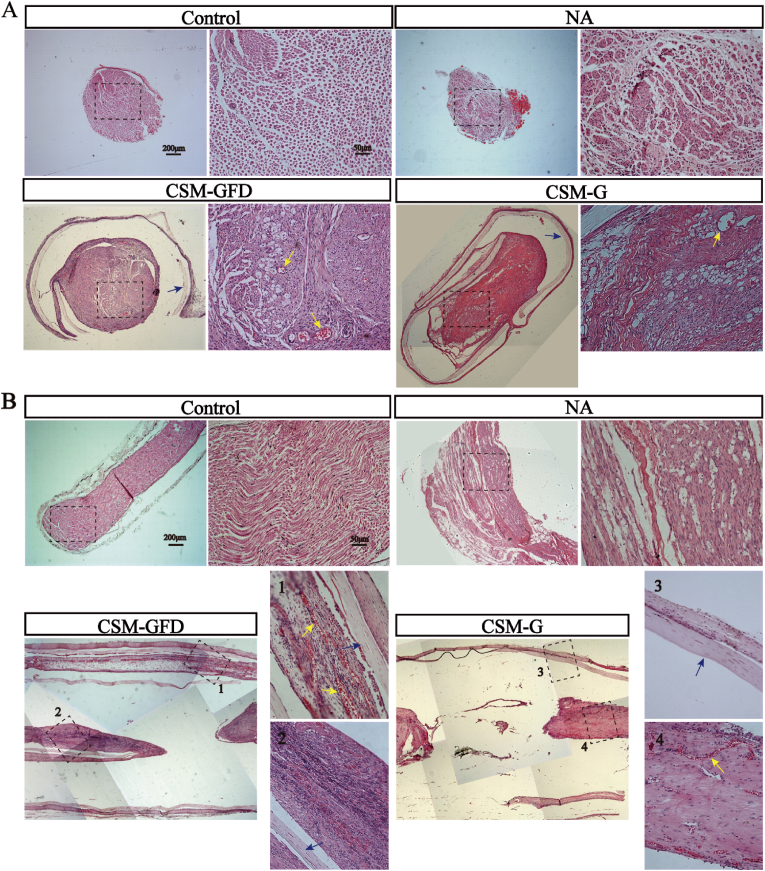
The HE images of cross-sections and longitudinal-sections of the regenerative nerve fibers after 12 weeks surgery. (A) The nerve conduit was cross sectioned in the middle to assess the regenerative nerves. (B) The nerve conduit was split longitudinally in the middle to observe the regenerated nerve fibers. Blue arrow: CSM conduit; Yellow arrow: newly regenerated blood vessels. (For interpretation of the references to colour in this figure legend, the reader is referred to the Web version of this article.)

12 weeks post-surgery, cross-section and longitudinal-section of implanted nerve graft and conduit was stained with HE and compared with normal nerve fiber at the same location in the shame control animal (Fig. 9). As shown in Fig. 9A, cross-sectional view of the control sciatic nerve displayed as a healthy and normal arrangement of nerve bundles and vessels with sheath wrapping the nerve fiber. In NA group, grafted nerve fiber showed similar nerve bundle arrangement as the control group did except a richer blood supply. Cross sectional view of the CSM-G and CSM-GFD groups was featured by a continuous outer structure of the conduit, which was encapsulated by a thin layer of connective tissue. The HE section of the conduit proved that a complete protection and guidance could be delivered by current design of tube. CSM-GFD group showed typical cross-sectional view of nerve bundle like structures neighboring to ample blood vessels. CSM-G group showed less nerve bundle like structures and blood vessels.

The HE images of longitudinal-sections of regenerative nerve fibers were displayed in Fig. 9B. In control and NA groups, nerve fibers were regularly arranged in bundles. In CSM-GFD group, lots of regeneration axon like cells and new blood vessels filled the conduit. From the mosaic histology photo of CSM-GFD group, an intact 3.66 mm long tissue containing nerve fiber like structure and blood vessel was successfully sectioned and retained during sample preparation. Considering the 3D porous architecture of hydrogel where DPSCs started to expand from, it was extremely difficult to longitudinally dissect single nerve fiber or tissue bundle in one dimension. In CSM-G group, a few regenerative nerve fibers run along the long axis in the background of matrix materials. Compared to CSM-GFD group, there was much less solid cell islands or tissue parts inside the conduit. The results were consistent with cross-sections images, indicating that CSM-GFD group generated more nerve like tissue and support tissue than CSM-G group did.

### 3.8 Toluidine blue staining

**Fig. 10:**
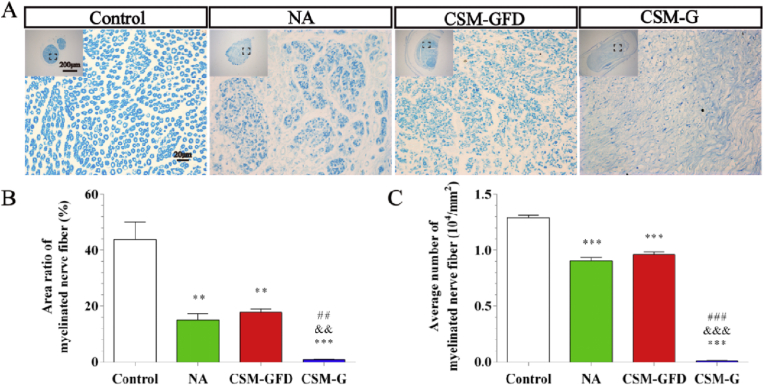
Toluidine blue staining of regenerated nerve from cross section of the middle of the transplanted tissue. (A) Light microscopy images of control, NA, CSM-GFD, and CSM-G groups. (B) Statistical analysis of the total area of myelinated nerve fibers. (C) Statistical analysis of the number of myelinated nerve fibers. ^∗^P < 0.05, ^∗∗^P < 0.01, ^∗∗∗^P < 0.001 versus the control group; ^&^P < 0.05, ^&&^P < 0.01, ^&&&^P < 0.001 versus the NA group; ^##^P < 0.01, ^###^P < 0.001 versus the CSM-GFD group. (For interpretation of the references to colour in this figure legend, the reader is referred to the Web version of this article.)

The myelin sheaths of regenerated nerves were evaluated by toluidine blue staining assay. As shown in Fig. 10A, cross section of the middle of the transplanted tissue showed regenerated myelinated nerve fibers with varied densities and numbers in each group. 12 weeks after the surgery, the total area ratio of myelinated nerve fibers in each group ordered as follow: control > CSM-GFD > NA > CSM-G (Fig. 10B). The total number of myelinated nerve fibers was highest in the control group and lowest in the CSM-G group (Fig. 10C). There was no significant difference of the total number of myelinated nerve fibers between the CSM-GFD group and NA group (P > 0.05).

**Fig. 11:**
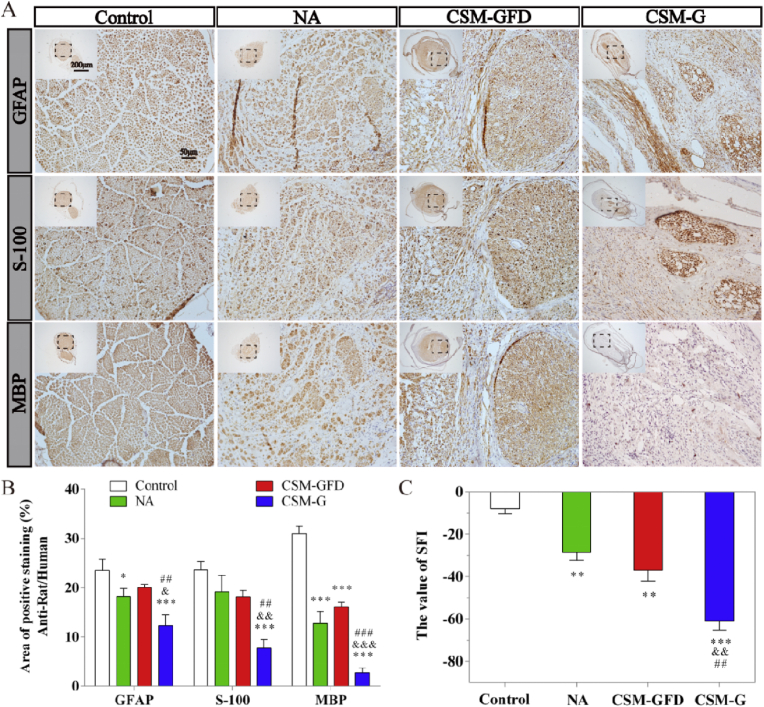
The analyses of immunohistochemistry and walking track test after 12 weeks surgery. (A) Representative images of the anti-rat/human GFAP, S-100 and MBP from immunohistochemisty. (B) Quantification of the anti-rat/human GFAP, S-100 and MBP positive staining ratio to normal in regenerated nerves. (C) The sciatic function index (SFI) of the control group and experimental groups. The quantification results obtained by Image J. Data were presented as mean ± standard error from 3 rats in each group. ^∗^P < 0.05, ^∗∗^P < 0.01, ^∗∗∗^P < 0.001 versus the control group; ^&^P < 0.05, ^&&^P < 0.01, ^&&&^P < 0.001 versus the NA group; ^##^P < 0.01, ^###^P < 0.001 versus the CSM-GFD group.

### 3.9 Immunohistochemical (IHC) and western blot (WB) analyses

As shown in the IHC analysis of the anti-rat/human neural specific markers (Fig. 11A and B), the expressions of neural specific markers in the NA and CSM-GFD groups showed stronger than those in the CSM-G group. In NA group, S-100 and GFAP was highly expressed, and MBP was moderately expressed. In CSM-GFD group, MBP, GFAP and S-100 were all highly expressed. All there neural special markers were weakly expressed in CSM-G group (Fig. 11A). In the 3 experimental groups, the intensity of GFAP positive regions was CSM-GFD > NA > CSM-G. The intensity of S-100 positive regions: NA > CSM-GFD > CSM-G. The intensity of MBP positive regions: CSM-GFD > NA > CSM-G. group (Fig. 11B).

**Fig. 12:**
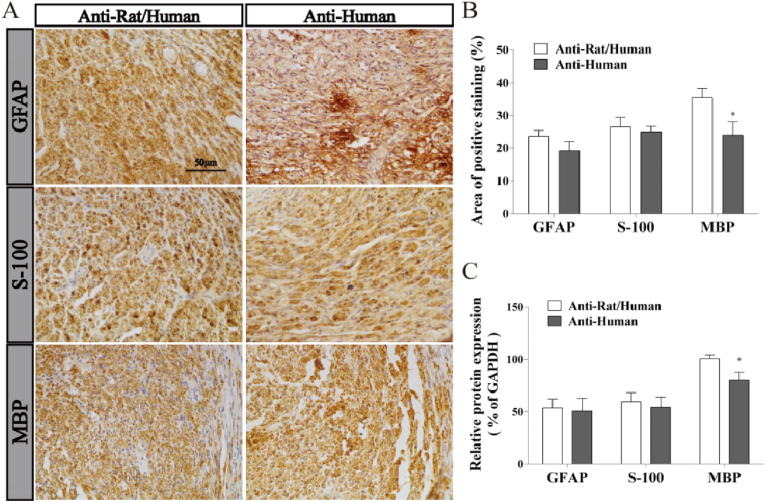
The analyses of immunohistochemistry and Western blot in CSM-GFD group after 12 weeks surgery. (A) Representative images of the anti-rat/human and anti-human GFAP, S-100 and MBP from immunohistochemisty. (B) The intensity of anti-rat/human and anti-human GFAP, S-100 and MBP positive staining. (C) Quantification of the anti-rat/human and anti-human GFAP, S-100 and MBP protein expression levels. The quantification results obtained by Image J. Data were presented as mean ± standard error from 3 rats in each group. ^∗^P < 0.05 versus the anti-rat/human group.

Recent study showed that in vivo transplanted MSCs such as DPSCs could indirectly assist endogenous nerve cells differentiation through the paracrine effects or directly differentiate into nerve cells in injured nerve [41]. In our study, the anti-human neural specific markers were evaluated by IHC and WB in CSM-GFD group, in order to confirm the effects of exogenous DPSCs (Fig. 12). As shown in IHC analysis (Fig. 12A), compared to the positive expression of the anti-rat/human neural specific markers, the anti-human neural specific markers MBP, GFAP and S-100 were also strongly expressed in CSM-GFD group. Meanwhile, the intensity of positive staining regions between the anti-rat/human and anti-human markers GFAP and S100 had no significant difference. However, the intensity of the anti-human MBP positive regions was lower than that of the anti-rat/human MBP marker, and the difference had statistically significant (Fig. 12B). The protein expressions of neural special markers MBP, GFAP and S-100 were detected by WB analysis. The results showed that the proteins of the anti-rat/human and anti-human MBP, GFAP and S-100 were all expressed in CSM-GFD group. The level of anti-human GFAP and S-100 expressions had no significant difference compared to those of anti-rat/human markers expressions. In CSM-GFD group, almost 100% of the positive expression of GFAP and S-100 and nearly 80% of the positive expression of MBP was from the anti-human neural special markers (Fig. 12C).

## 4 Discussion

Peripheral nerve injuries, which caused by trauma, tumor and iatrogenic injury, influence 130 to 230 per million people each year [50]. Treating peripheral nerve injuries with large defect is one of the most challenging problems in clinic. As for the peripheral nerve repair strategy, autografts consider the “gold standard” and provide the best opportunity for bridging larger gaps of nerve injuries. There are some drawbacks in the use of autograft, such as donor site morbidity and loss of function, infection, formation of painful neuroma, and extending recovery time [51]. Therefore, these limitations of autograft urge the develop of other approaches such as bio-engineering novel nerve conduit to repair large gap in peripheral nerve injuries.

The development of nerve conduit had gone through three main stages: the first generation of hollow nerve conduits prepared from non-resorbable materials (e.g. silicone and polytetraﬂuoroethylene). This hollow design was able to guide the regeneration of peripheral nerve and form a barrier against the infiltration of connective tissue. Yet there were some limitations of this hollow conduits, such as causing secondary surgery, chronic tissue response, and nerve compression [4,7]. The second generation of nerve conduits fabricated from resorbable materials such as collagen, polyglycolic acid and poly-dl-lactide-co-caprolactone, which had good biocompatibility and degradability. In addition, they had the ability to load growth factor for a prolonged release. This second generation of conduit has been widely used in the repair of nerve defects. But they seldomly promoted a functional recovery to injured nerve [7]. Hence, the third generation of nerve conduit, aiming to address this functional recovery issue, included scaffolds, stem cells, controlled release of growth factors, and extracellular matrix proteins to provide a promising strategy to bridge large gap in nerve injuries [52].

In this study, we used cellulose/soy protein isolate composite membranes (CSM) as the hollow conduit, which had good mechanical behavior and great biocompatibility, biodegradability and permeability. CSM, a second generation conduit, has been proved as a suitable scaffold material in peripheral nerve tissue engineering [7]. Unfortunately, a single hollow conduit couldn't fulfill the requirements for nerve regeneration due to the limited inner surface areas for cell growth and attachment, as well as the inability to provide the ECM-like structures for regenerated nerves to reconstruct the optimal native spatial arrangement within the conduit [53]. Nowadays, biomimetic nerve conduits, which was based on hollow conduits incorporated with intraluminal fillers such as fibers, sponges and hydrogels, had been developed to overcome these limitations [54]. Among these fillers, hydrogels, having 3D network structures with and retaining 80% water, had great biocompatibility and biodegradability. Hydrogel could simulate ECM-like structures to provide a suitable microenvironment for cell to grow and proliferate as well as to encapsulate bioactive growth factors to achieve ae sustained-release profile. Various type of hydrogels have been wildly tested in nerve tissue engineering [2,19,55].

In this study, GelMA hydrogels containing bFGF (GelMA-bFGF) was used as the intraluminal fillers. It possessed ECM-like porous structure and optimal physi-chemical properties (Fig. 2, Fig. 3, Fig. 4, Fig. 5). The microstructure of hydrogels played a crucial role in physical and biological properties. Study has confirmed that hydrogel microstructure could influence cellular infiltration and survival [56]. Compared to the large pores of GelMA hydrogels, GelMA-bFGF hydrogels contained two types of porous structure: large pores and internally connected small pores (Fig. 2A). The later had higher surface area and might enhance the exchange of nutrients and the excretion of cellular metabolites. Meanwhile, the pore sizes of both GelMA and GelMA-bFGF hydrogels decreased with the increase of GelMA contents (Fig. 2B and C). Previous studies revealed that factors like GelMA concentration, degree of methacryloyl substitution, UV exposure time and initiator concentration were major parameters of the synthesis and processing of hydrogels have been shown to affect the physical properties of GelMA hydrogels [26,29]. As GelMA concentration increased, more chemical cross-link per unit volume could be formed, which would enhance the pore sizes of hydrogels to become smaller. In our work, the results indicated that more cross-link exhibited in both GelMA and GelMA-bFGF hydrogels in high GelMA contents.

The swelling ability, an important feature of the hydrogels, is related to the pore structure, degree of crosslinking and total polymer. It affects the diffusion of nutrients, oxygen, and metabolic waste [26,57]. Our results indicated that the swelling ratio of 5%, 10% and 15% GelMA-bFGF hydrogels gradually decreased with an increase of GelMA contents (Fig. 3A). The results might be attributed to the relative large pore size in lower GelMA proportion of hydrogels, which facilitated the solutions filling into the interconnected pores of hydrogels [58]. In addition, the swelling ratio of GelMA-bFGF hydrogels decreased by increasing the total polymer concentrations, which was consistent with the observation on pure GelMA hydrogels described in previous study [28].

Moreover, GelMA hydrogels had the ability of a sustained-release of growth factors. In our study, the release rate of bFGF in GelMA-bFGF hydrogels decreased as the increase of GelMA concentration in the hydrogels (Fig. 3C). This result might be attributed to a larger intermolecular space and pore size in lower concentration of GelMA in the hydrogels, which accelerated the release of bFGF [33]. Biodegradability is an important physical property of hydrogels providing sufficient space for newly regenerated tissue. Our in vitro and in vivo results indicated that the degradation rate of GelMA-bFGF hydrogels increased with the decrease of GelMA contents in the hydrogels (Fig. 4, Fig. 5). We assumed that due to lower degree of cross-link presented in lower GelMA concentration of the hydrogels, large size porous structure and great swelling ability facilitated the collagenase to access into the center of hydrogels and thus accelerated its degradation [57].

As for the biocompatibility, dental pulp stem cells (DPSCs), originated from the cranial neural crest and expressed many neural markers, have been widely explored as seed cells in nerve regeneration [34,35]. In this study, we demonstrated that GelMA-bFGF hydrogels was highly compatible with DPSCs, which provided an appropriate microenvironment for DPSCs survival and growth. DPSCs continued to proliferate from day 1 to day 9 in three concentrations of GelMA-bFGF hydrogels with DPSCs proliferating much better in 5% and 10% GelMA-bFGF hydrogels than in 15% GelMA-bFGF hydrogel (P < 0.05) (Fig. 6). This might be attributed to the larger pore sizes, higher swellability and faster degradability existed in the 5% and 10% hydrogels, which enhanced nutrients infiltration and metabolic waste diffusion.

According to above results, 10% GelMA-bFGF hydrogel displayed optimal physical characteristics and biological properties. When compared with 10% pure GelMA hydrogel, 10% GelMA-bFGF hydrogel served as a better scaffold, in which DPSCs proliferated more and spread more widely (Fig. 7, Fig. 8). The results indicated that bFGF, a growth factor, could significantly promote DPSCs survival and proliferation, which was consistent with the findings in previous study [45]. Therefore, 10% GelMA-bFGF hydrogel was chosen as the intraluminal fillers of our third generation nerve conduit for the regeneration of large gap peripheral nerve injuries.

In this study, we constructed the third-generation of nerve conduits by filling CSM hollow conduit with 10% GelMA-bFGF hydrogel loaded with DPSCs. The in vivo effects of this novel nerve conduit on nerve repair and regeneration were comparatively evaluated by SFI, histological, immunohistochemical and Western Blot analyses. To assess the neural regeneration effect of GelMA-bFGF hydrogels with DPSCs in long gap nerve defect, animals were divided into 4 groups including sham control group, nerve autograft (NA) group, CSM nerve conduit filled with GelMA hydrogel (CSM-G) group, and CSM nerve conduit filled with GFD hydrogels (CSM-GFD) group. A 15-mm nerve gap was on sciatic nerve (Fig. A2) and nerve repair outcome was studied after 12 weeks post-surgery. All animals were tolerate to the surgery and survived through recovery period till humane end point.

SFI, the sciatic functional index, was performed to evaluate the motor function of the experimental animals. Previous studies scaled the repair effect of normal to total impairment using SFI value from 0 to −100 [7,59]. Our results confirmed that CSM-GFD, GelMA-bFGF combined with DPSCs strategy, greatly promoted the functional repair of peripheral nerve injuries (Fig. 11C).

In Fig. 9, HE slides of repaired defect showed that more new nerve fibers and blood vessels in the middle of the transplanted section were observed in CSM-GFD group, in which a 3.66 mm long continuing newly formed nerve fiber was captured in longitudinal HE slicing. And these newly formed nerve fibers in CSM-GFD group were often myelinated according to toluidine blue staining assay (Fig. 10), which had similar total area and number of myelinated nerve fibers to those in NA group, the golden standard in nerve grafting. In CSM-G group, lacking growth factor and stem cells, only few regenerated nerve fibers were presented in cross sections of the transplanted portion and the nerve fibers were less myelinated. These results worked complementary with SFI study suggesting that DPSCs and bFGF were suitable for nerve fiber regeneration, as previously described [45].

IHC analysis on regenerated nerve tissue using anti-rat/human neural specific markers provided an in-depth observation on the cell type identification of regenerated cells (Fig. 11A and B). GFAP and S-100 were the Schwann-like cell phenotype markers, which indicated the regeneration of axonal fibers, as well as MBP was the myelin specific marker, meaning the newly generated myelin sheath [41]. In our study, we found that the regenerated nerve tissue positively expressed all three markers in CSM-GFD group, like the NA group, the golden standard (Fig. 11B). Hence, we claimed that CSM-GFD could regenerate the two types of nerve cells, axon and Schwann like cells. This finding was echoed by functional study, SFI, and histology study, toluidine blue staining assay.

Current research on DPSCs speculated that they could promote the in vivo nerve regeneration through both indirectly assisting the endogenous nerve cell differentiation and direct differentiating into nerve cells [41]. In this work, to investigate the origin of newly regenerated nerve cells, we used anti-human neural specific markers of GFAP, S-100 and MBP to stain the new nerve tissue. We found three anti-human neural markers were positively expressed in CSM-GFD group (Fig. 12). Nearly 100% GFAP and S-100 was from the anti-human neural special markers, and about 80% MBP came from the anti-human neural special markers. In this case, our results verified that it was the exogenous DPSCs that directly differentiated into new axons fibers and myelin sheaths in the repair of a large gap of peripheral nerve injuries.

Taken together, we have experimented an optimal design of the conduit, CSM-GFD. Cellulose/soy protein isolate composite membranes (CSM) formed a hollow conduit tube that provided space to accommodate scaffold material, prevent scar tissue formation and provide a guiding bridge for the newly generated nerve spreading from proximal end to distal end. GFD was the combination of 10% GelMA-bFGF with DPSCs. 10% GelMA hydrogel had 3D porous architecture and cell friendly microenvironment mimicking an ECM-like structure, it could facilitate cell adhesion, survival and growth and provided a sustained release of growth factors [26]. DPSCs, derived from the cranial neural crest lineage, preserved an outstanding potential for neuronal differentiation, and also expressed multiple factors which were suitable for neural repair and regeneration. DPSCs could also expressed immunomodulatory factors that stimulated the formation of blood vessels and enhanced the regeneration and repair of injured nerve [2]. In this study, our results indicated that DPSCs differentiated into neuron and Schwann like nerve cells and formed myelinated nerve fibers. Moreover, most newly formed nerve tissue was directly from DPSCs rather than endogenous nerve cells. Basic fibroblast growth factor (bFGF) was able to promote cell survival and proliferation, and also had beneficial effect on neural regeneration and functional recovery after nerve injuries [45].

## 5 Conclusions

In conclusion, this third-generation nerve regeneration conduit achieved functional recovery to the same level as the nerve autograft could. The design of CSM-GFD was able to repair a large gap (15 mm long) of peripheral nerve verified in a rat model. We confirmed the regenerated nerve tissue was mainly contributed by the exogeneous DPSCs in this CSM-GFD conduit. Our nerve regeneration conduit, consisting of 10% GelMA-bFGF and DPSC that were wrapped by a cellulose/soy protein isolate composite membrane, was proved to be a promising tissue engineering approach to treat the large gap defect in peripheral nerve injuries.

## CRediT authorship contribution statement

**Lihua Luo:** Formal analysis, Writing - original draft. **Yan He:** Formal analysis, Writing - original draft, Writing - review & editing. **Ling Jin:** Formal analysis, Writing - original draft. **Yanni Zhang:** Formal analysis. **Fernando P. Guastaldi:** Writing - review & editing. **Abdullkhaleg A. Albashari:** Methodology. **Fengting Hu:** Methodology. **Xiaoyan Wang:** Methodology. **Lei Wang:** Methodology. **Jian Xiao:** Supervision. **Lingli Li:** Supervision. **Jianming Wang:** Supervision. **Akon Higuchi:** Supervision. **Qingsong Ye:** Conceptualization, Funding acquisition, Supervision.

## Declaration of competing interest

The authors declare that there is no known conflict of interest that could influence the findings reported in this paper, neither at personal nor at organizational level.
