# Supplementary material for: Application of bioactive hydrogels combined with dental pulp stem cells for the repair of large gap peripheral nerve injuries
Source: Bioact Mater. 2020 Sep 19;6(3):638–54. doi: 10.1016/j.bioactmat.2020.08.028 (PMC7509005; doi:10.1016/j.bioactmat.2020.08.028)
Supplement: Multimedia component 1 [file mmc1.doc]

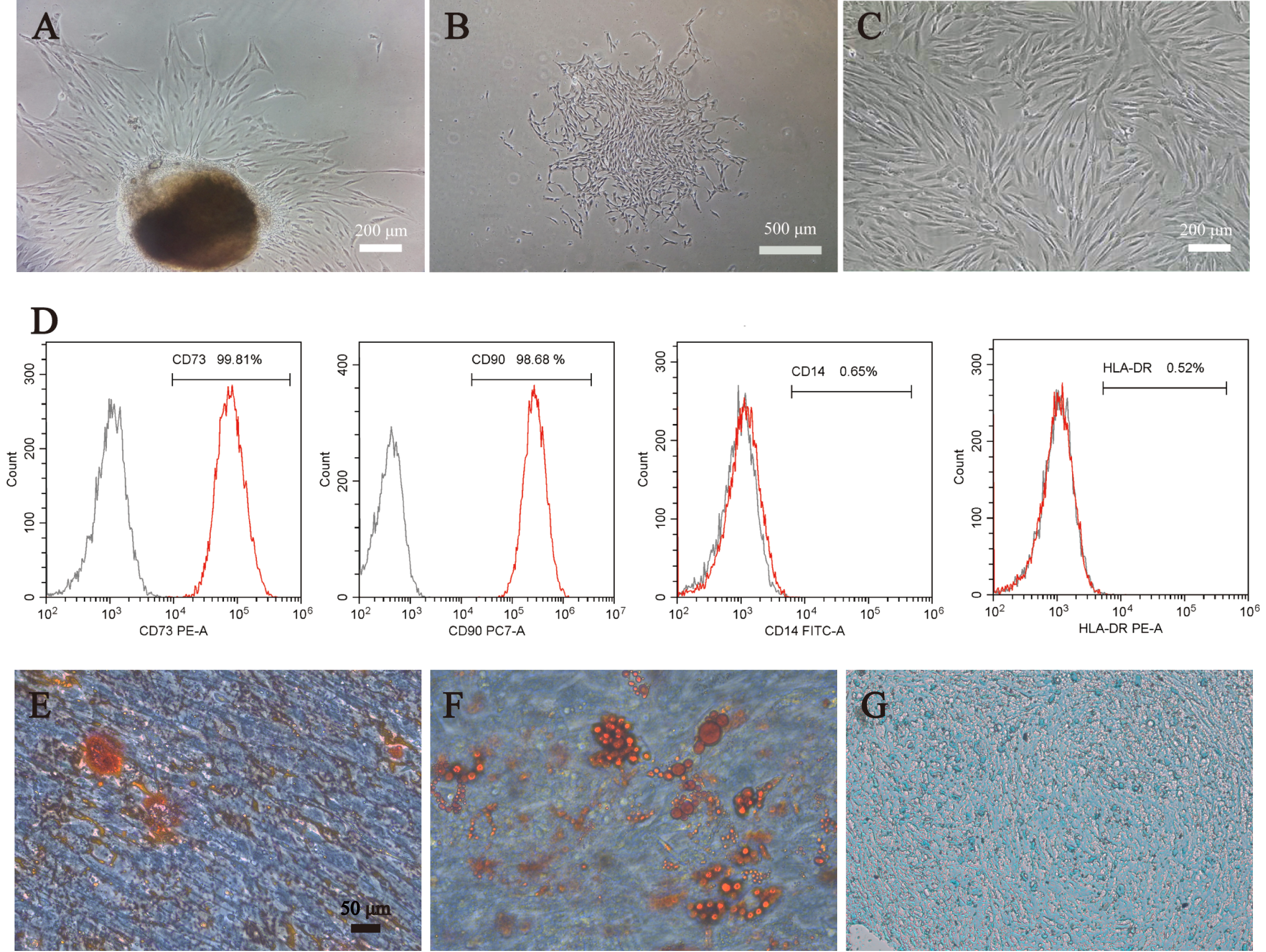


**Supplementary Fig. A1.** Isolation, culture, and identification of DPSCs. (A) (B) and (C) DPSCs culture at day 7, day 10, and the first passage, respectively. (D) The expression of surface markers of DPSCs. (E) Osteogenic differentiation, (F) Adipogenic differentiation, and (G) Chondrogenic differentiation.


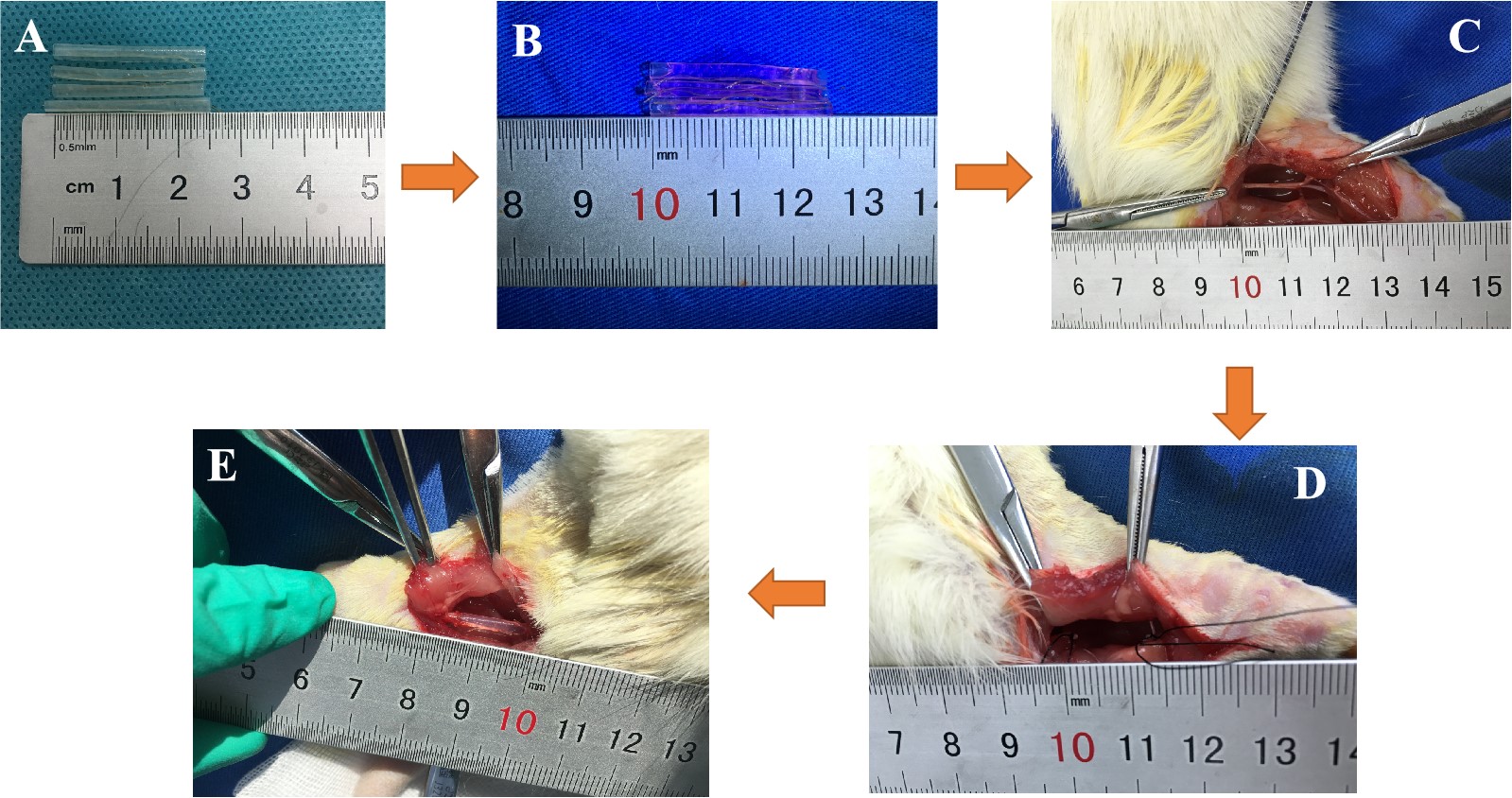


**Supplementary Fig. A2.** The procedure of animal experiment. (A) CSM hollow conduit. (B) CSM-GFD nerve conduit. (C) Sciatic nerve. (D) 14-15 mm long gap of sciatic nerve. (E) Bridging of the nerve defect.
